# Identification of Parkinson’s disease using MRI and genetic data from the PPMI cohort: an improved machine learning fusion approach

**DOI:** 10.3389/fnagi.2025.1510192

**Published:** 2025-02-04

**Authors:** Yifeng Yang, Liangyun Hu, Yang Chen, Weidong Gu, Guangwu Lin, YuanZhong Xie, Shengdong Nie

**Affiliations:** ^1^Department of Medical Imaging, Huadong Hospital, Fudan University, Shanghai, China; ^2^Center for Functional Neurosurgery, RuiJin Hospital, Shanghai Jiao Tong University School of Medicine, Shanghai, China; ^3^School of Health Science and Engineering, University of Shanghai for Science and Technology, Shanghai, China; ^4^Department of Anesthesiology, Huadong Hospital, Fudan University, Shanghai, China; ^5^Medical Imaging Center, Taian Central Hospital, Shandong, China

**Keywords:** Parkinson’s disease, imaging genomics, stable feature selection, multi-modal fusion, machine learning

## Abstract

**Objective:**

This study aim to leverage advanced machine learning techniques to develop and validate novel MRI imaging features and single nucleotide polymorphism (SNP) gene data fusion methodologies to enhance the early identification and diagnosis of Parkinson’s disease (PD).

**Methods:**

We leveraged a comprehensive dataset from the Parkinson’s Progression Markers Initiative (PPMI), which includes high-resolution neuroimaging data, genetic single-nucleotide polymorphism (SNP) profiles, and detailed clinical information from individuals with early-stage PD and healthy controls. Two multi-modal fusion strategies were used: feature-level fusion, where we employed a hybrid feature selection algorithm combining Fisher discriminant analysis, an ensemble Lasso (EnLasso) method, and partial least squares (PLS) regression to identify and integrate the most informative features from neuroimaging and genetic data; and decision-level fusion, where we developed an adaptive ensemble stacking (AE_Stacking) model to synergistically integrate the predictions from multiple base classifiers trained on individual modalities.

**Results:**

The AE_Stacking model achieving the highest average balanced accuracy of 95.36% and an area under the receiver operating characteristic curve (AUC) of 0.974, significantly outperforming feature-level fusion and single-modal models (*p* < 0.05). Furthermore, by analyzing the features selected across multiple iterations of our models, we identified stable brain region features [lh 6r (FD) and rh 46 (GI)] and key genetic markers (rs356181 and rs2736990 SNPs within the SNCA gene region; rs213202 SNP within the VPS52 gene region), highlighting their potential as reliable early diagnostic indicators for the disease.

**Conclusion:**

The AE_Stacking model, trained on MRI and genetic data, demonstrates potential in distinguishing individuals with PD. Our findings enhance understanding of the disease and advance us toward the goal of precision medicine for neurodegenerative disorder.

## 1 Introduction

Parkinson’s disease (PD) is marked by the degeneration of dopamine-producing neurons in the substantia nigra, and the accumulation of alpha-synuclein protein in the midbrain. While PD is predominantly sporadic, genetic factors are increasingly recognized in its development. Numerous studies highlight the importance of genetics, identifying genes such as SNCA, LRRK2, PINK1, and GBA, which are linked to both dominant and recessive forms of inherited PD (Blauwendraat et al., 2020; Fernandez-Santiago and Sharma, 2022; Tranchant, 2019). These genes play crucial roles in maintaining cellular health, and disruptions can lead to neurodegeneration, affecting symptoms, onset age, and disease progression (Alfradique-Dunham et al., 2021; Chu et al., 2009; Davis et al., 2016; Iwaki et al., 2019). Thus, monitoring gene expression changes is vital for early diagnosis and prediction of PD.

The fusion of genetic and imaging data has emerged as a promising approach to understanding the interplay between genetic predispositions and brain structure in PD. Recent studies have highlighted that combining genetic and neuroimaging markers can significantly enhance predictive and diagnostic accuracy for PD (Kim et al., 2017; van Nuenen et al., 2009; Won et al., 2019; Won et al., 2020). For example, Kim et al. utilized a linear regression model incorporating single nucleotide polymorphism (SNP) genetic features and structural connectivity data to predict clinical scores on the Movement Disorder Society-Unified Parkinson’s Disease Rating Scale (MDS-UPDRS). Their model demonstrated superior predictive performance, with a correlation coefficient (between model prediction outcomes and actual MDS-UPDRS score) of 0.788 (Kim et al., 2017). Won et al. employed the Lasso algorithm to select key features from structural connectivity and SNP genetic data, constructing a linear regression model to predict Geriatric Depression Scale (GDS) scores. This model exhibited a meaningful correlation (*r* = 0.749) between predicted and actual GDS scores (Won et al., 2019). Building on these findings, the same team applied sparse typical correlation analysis to integrate imaging genetic features, achieving a correlation of *r* = 0.5486 between predicted and actual ages at PD onset (Won et al., 2020). These studied underscores the feasibility of predicting disease onset by fusing multi-modal data.

For individual PD diagnosis, Xia et al. developed a clustered evolutionary random neural network model to analyze fused functional magnetic resonance imaging (fMRI) and SNP data. This model achieved an impressive accuracy of 88.57% in identifying PD patients and uncovered additional PD-related genes and brain regions (Bi et al., 2021a). Lei et al. proposed a joint learning framework based on MRI features using a multi-branch octave convolutional neural network (FMOCNN) to diagnose PD in gene-related cohorts. The accuracy of this method in identifying individuals with genetic cohort PD (GenPD) and genetic mutation but not PD cohort (GenUn) was 84.91% (Lei et al., 2022). The fusion of multimodal information from images and genes leverages complementary data, providing a more comprehensive characterization of the pathological mechanisms of PD. This integrated approach offers a new perspective for the diagnosis and prediction of PD.

Machine learning (ML) techniques offer a robust method for processing large and complex genome-wide SNP datasets (Makarious et al., 2022; Szymczak et al., 2009). However, analyzing genomic data poses significant challenges due to its high-dimensional nature, where the number of features usually far exceeds the number of samples. This high-dimensionality engenders a plethora of redundant information, which would lead to multicollinearity among the high-dimensional genetic variables, complicating model training. This complexity can lead to multicollinearity among key variables, complicating model training. Limited sample sizes further increase the risk of overfitting, even with regularization methods (Pudjihartono et al., 2022). Overcoming the challenges of “curse of dimensionality” is essential for developing accurate predictive models from genomic data.

Our aim is to improve early PD identification by leveraging features from high-dimensional imaging and genetic data. This involves addressing differences between genetic and imaging data to enable advanced multimodal integration. Firstly, we preprocess MRI and SNP gene data separately and extract relevant features. We then developed two multimodal data fusion methods, feature-level and decision-level fusion, to fuse them. For feature-level fusion, we employ a combined feature selection method called Fisher-EnLasso-PLS, which enables collaborative analysis of multimodal data, effectively reducing data complexity while more accurately capturing key features associated with the disease. For decision-level fusion, we develop an AE_Stacking model to enhance the integration of image and genetic features. This approach maintains the multidimensional and complex nature of diseases. By integrating multiple data sources and models, it could enhance prediction accuracy and robustness, minimizing potential error from relying on a single data source or single model.

## 2 Materials and methods

### 2.1 Participants

The data in this study accessed from the Parkinson’s Progression Markers Initiative (PPMI) database,^1^ a multicenter database that includes neuroimaging, gene data such as single-nucleotide polymorphisms (SNP) and relevant clinical information of various PD individuals and matched healthy controls. Inclusion criteria for individuals of the early PD cohort were baseline participants diagnosed with PD for two years or less, whose DAT scans indicated a dopaminergic deficit, and who had not commenced any medication. For the health controls (HC), they had never been diagnosed with any major neurological disorder and had no first-degree relatives with idiopathic PD.

#### 2.1.1 Clinical neuropsychological assessments

Patients with PD were assessed using the Unified Parkinson’s Disease Rating Scale (UPDRS) part III examination scale (range 0–108), and Hoehn & Yahr scale (H&Y) stage I & II. For each subject, several available clinical neuropsychological evaluation scales were also used: the University of Pennsylvania Smell Identification Test (UPSIT), Geriatric Depression Scale (GDS), Montreal Cognitive Assessment (MOCA), and Scales for Outcomes in Parkinson’s Disease—Autonomic Dysfunction (SCOPA-AUT).

#### 2.1.2 SNP data acquisition

Subjects’ genotyping SNP information was collected on the NeuroX genotyping chip (Ghani et al., 2015; Nalls et al., 2015). The NeuroX array is an Illumina Infinium iSelect HD Custom Genotyping array containing 267,607 Illumina standard contains exonic variants and an additional 24,706 custom variants designed for neurological disease studies. The content of the NeuroX is available on the PPMI site.^2^

#### 2.1.3 MRI acquisition

For data consistency, only 3T MRI scanner (SIEMENS MAGNETOM Trio scanner) with high-resolution MPRAGE T1 sequence was chosen with the following parameters: repetition time (TR) = 2300.0 ms, echo time (TE) = 3.0 ms, Inversion time (TI) = 900.0 ms, flip angle = 9.0 degree, and slicing thickness = 1.0 mm. Participants with missing images or incomplete scans were excluded from the study. A total of 209 subjects (135 PD patients and 74 HC) were final downloaded.

### 2.2 Data preprocessing

#### 2.2.1 SNP data selection, cleaning, and quality control

In this paper, genotyping data were collected from the PPMI dataset for a total of 619 subjects, each containing 267,607 SNP loci. Quality control of SNP data was performed using Plink v1.09 software and referring to the ENIGMA protocol (Purcell et al., 2007; Thompson et al., 2022). The quality control of SNP data included sample quality control and SNP locus quality control, as follows:

Sample quality control: (1) sample detection rate detection; (2) sex check; (3) sibling pair identification; (4) population stratification;

Quality control: (1) minor allele frequency (MAF) < 1%; (2) genotype call rate < 95%; (3) Hardy-Weinberg equilibrium (HWE) test *p* < 10-6.

SNP loci that did not meet the criteria were excluded. After rigorous quality control, a total of 532 subjects (367 PD patients and 165 HC) were retained, each of which contained 48,414 high-quality SNP locus information for use in subsequent experiments.

#### 2.2.2 MRI data processing and feature extraction

A computational anatomy toolbox (CAT 12.7-r1727)^3^ based on statistical parametric mapping software (SPM 12)^4^ was used to preprocess the data, including voxel-based morphometry (VBM) and surface-based morphometry (SBM) analysis (Supplementary Figure S1). As a supplement to the VBM, the SBM allows the calculation of multiple GM tissue features at varying scales (Farokhian et al., 2017; Seiger et al., 2018). The standardized structural processing pipeline includes head motion correction, MR field inhomogeneity correction, brain extraction, automated segmentation of GM, WM, and cerebrospinal fluid (CSF) (WMH corrected), topological defect correction, cortical surface reconstruction, and tessellation of the boundary between the WM and cortical GM. Cortical morphological parameters including cortical thickness (CT), surface area (SA), fractal dimension (FD) and gyrification index (GI) were calculated to quantify the local microstructural changes of brain GM structure (Luders et al., 2006; Sandu et al., 2014; Wang et al., 2021). Next, the template-based matching method was applied to define regions of interest (ROIs) and to extract multiple structural morphological parameters from ROIs. For this study, spatially matching the GMV map with Brainnetome atlas could determine the volume of the 246 subregions (Fan et al., 2016). Cortical areas were defined by the high-resolution Human Connectome Project multimodal parcellation (HCP-MMP1.0) (Glasser et al., 2016). Mean cortical thickness, surface area, thickness, fractal dimension and gyrification index were extracted from 360 cortical parcels in the HCP-MPP1.0. Finally, MRI data from 128 PD patients and 71 HC participants were retained after image quality control. There were total 1686 (246 + 360 × 4) extracted feature dimensions for each participant.

#### 2.2.3 SNP and MRI data merging

**FIGURE 1:**
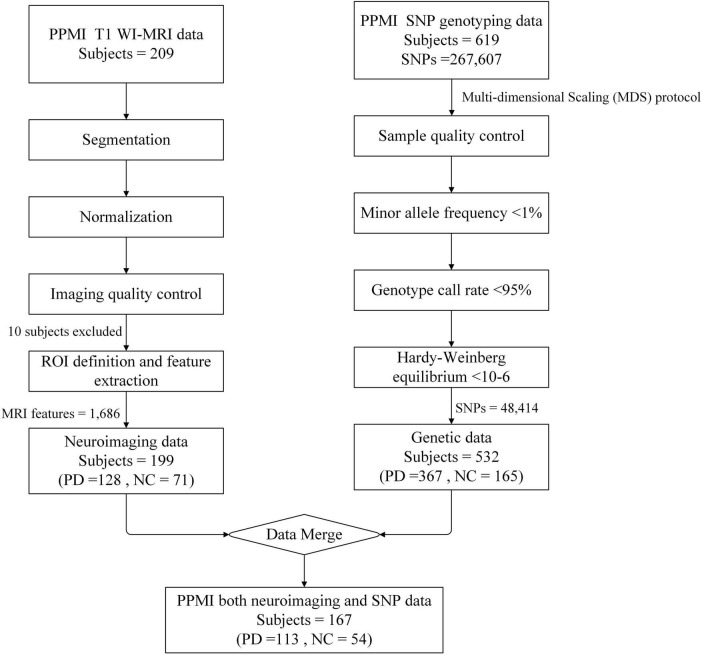
Schematic overview of data screening. For T1 WI-MRI, a total of 209 subjects were downloaded from the PPMI database, including 135 individuals with Parkinson’s disease (PD) and 74 health controls (HC). The images underwent preprocessing steps including segmentation and normalization, and were final registered to the MNI standard brain space. Following image quality control procedures, 7 PD subjects and 3 HC were excluded due to poor registration results. Ultimately, 128 PD and 71 HC subjects were retained for region of interest (ROI) definition and multidimensional MRI feature extraction. For genetic data, 619 subjects were downloaded from the PPMI database, including 383 PD and 178 HC, with 58 subjects’ phenotypes were missing. Firstly, based on the multi-dimensional scaling (MDS) protocol, 16 PD and 13 HC were excluded due to anomalies in the phenotype data. Subsequently, quality control was performed on the SNP sites, resulting in the exclusion of 219,193 SNPs that did not meet the quality criteria. Finally, a total of 167 subjects had both modalities of data, consisting of 113 PD patients and 54 HC, with each subject contributing 1,686 MRI features and 48,414 high-quality SNP data.

Samples with SNP genotype data were combined with samples with MRI imaging data. The final sample with data from both modalities consisted of 167 cases, including 113 early PD patients and 54 HC healthy controls (Figure 1). All data collection was approved by the relevant institutions, and participants signed a written informed consent.

### 2.3 Feature selection

A novel hybrid feature selection method (Fisher-EnLasso-PLS), which integrates Fisher discriminant analysis, lasso-based integrated stable feature selection algorithm and partial least squares (PLS) algorithm, was designed to optimize the feature selection process in high-dimensional imaging genetics data through phased refined feature screening strategy. Fisher discriminant analysis initially selects features that enhance class distinction, the Lasso-based algorithm selects robust features via sparsity, and the PLS algorithm captures the relationships between features and the response variable. By combining these techniques, this hybrid feature selection method could not only reduces dimensionality but also extracts essential information while minimizing the risk of model overfitting. The method is detailed below:

Firstly, Fisher discriminant analysis is employed to initially filter out irrelevant features. This method is efficient and computationally simple, demonstrating strong performance in high-dimensional gene feature selection (Sun et al., 2019; Zhang et al., 2021). A higher Fisher score indicates a feature’s capacity to differentiate between PD and HC samples.

Following this initial filtration, the number of candidate features is reduced to a more manageable level. However, it is important to note that Fisher’s method primarily emphasizes feature correlation and does not account for redundancy or interaction among features. To address this limitation, the ensemble Lasso (EnLasso) algorithm is applied next. EnLasso leverages data perturbation and ensemble learning to synthesize results from multiple training subsets, thus enhancing the accuracy and robustness of feature selection (Wang et al., 2022). Specifically, using stratified tenfold cross-validation, 90% of the training samples are selected and subsequently balanced through the SVM-SMOTE resampling method, which alleviates inaccuracies in feature selection stemming from class imbalances. The SVM-SMOTE method integrates the principles of SVM with synthetic data generation techniques; it leverages SVM to identify the boundary samples of the training set—support vectors—and performs adaptive interpolation within the kernel space on these support vectors to generate new samples, which has good performance in non-linear and high-dimensional data scenarios. The Lasso algorithm is then applied to these subsets, with training sample perturbations enhancing the stability of feature selection results. This process is repeated 10 times to produce M candidate feature subsets*S*_*i*_ (1 ≤ *i* ≤ *M*). An ensemble strategy then amalgamates these subsets by comprehensively assessing both feature occurrence frequency (*FS*_*q*_) and assigned weights (*FS*_*w*_), resulting in an importance score (IS) for each feature, calculated using the following formula:


(1) $$FS_{q}\left(f\right)=\frac{1}{M}\sum_{m=1}^{M}1\left\{f\in S\left(D_{i}\right)\right\}$$

where $S\,\left(D_{i}\right)=\left\{f:\omega_{f}^{\left(i\right)}\neq 0\right\}$ was defined as all the selected features performed by the feature selection algorithm on the *th i* sampled training subset *D*_*i*_. When the feature was selected, it was assigned 1, otherwise it was 0. *FS*_*q*_ metric quantifies the consistency with which a feature is selected across multiple iterations of the feature selection algorithm when applied to various training subsets. The higher the *FS*_*q*_, the stronger the stability of the feature itself.


(2) $$F\,S_{w}\,\left(f\right)=\frac{1}{M}\,\sum_{m=1}^{M}\omega_{f}^{D_{i}}$$

Where $\omega_{f}^{D_{i}}$ represented the weight assigned to the feature *f* when performing the feature selection algorithm on the *th i* sampled training subset *D*_*i*_. *FS*_*w*_ reflectes the feature’s contribution to accurately identifying the target variable. Features with higher weights are more influential in distinguishing between classes, thereby enhancing the model’s classification performance.


(3) $$I\,S\,\left(f\right)=\frac{1}{2}\,\left(F\,S_{q}+F\,S_{w}\right)$$

By integrating *FS*_*q*_ and *FS*_*w*_, we calculated the IS, which is a composite measure that encapsulates both the frequency of feature occurrence and the weights assigned to them. The IS ensures that the selected features are not only consistently chosen across different iterations but also have a substantialcontribuation on the model’s predictive capabilities. By sorting the features in descending order based on their IS values, a new ranked list of features is generated, from which the top-k features are selected using a forward search strategy.

In the final stage, to account for feature interaction, the PLS algorithm was employed for further dimensionality reduction, extracting principal components most pertinent to PD. Unlike PCA, which is an unsupervised method that considers only the characteristic variable data, PLS maximizes covariance between independent and dependent variables. It combines principal component analysis, correlation analysis, and regression not only addresses feature interactions to eliminate correlations among the data but also incorporates label vectors, effectively preserving key information related to the target task (Li et al., 2008).

### 2.4 Feature fusion

There are two kinds of feature fusion methods: feature level and decision level.

#### 2.4.1 Feature level fusion

**FIGURE 2:**
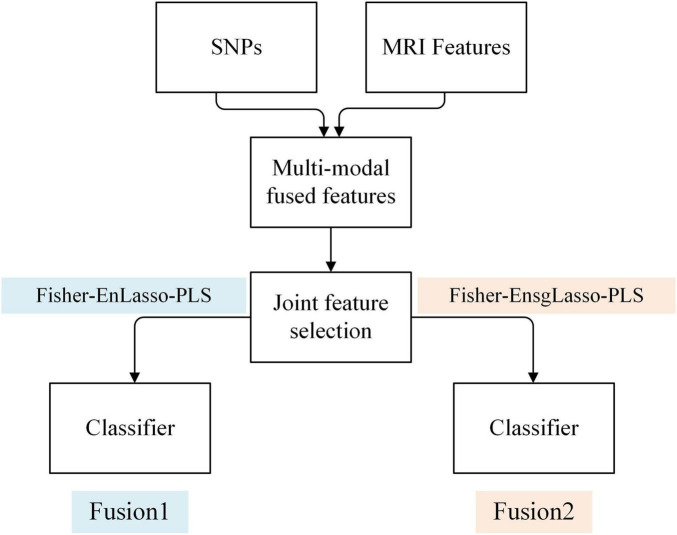
Framework of the fusion method of SNPs and MRI features based on feature level.

Feature-level multimodal fusion combines features from different modalities into a new matrix for joint feature selection and model training. This study merged genetic and MRI image features into a 50,100-dimensional matrix. To facilitate feature selection from this high-dimensional sparse data, we devised two optimization strategies, shown in Figure 2.

1. Fusion1

The first strategy employs the Fisher-EnLasso-PLS algorithm, as detailed in the *Feature Selection* section, for joint feature selection on the fusion matrix. Subsequently, model training is conducted to develop a multimodal fusion classification model, referred to as Fusion1.

1. Fusion2

Given the inherent differences between genetic and MRI modalities in characterizing Parkinson’s disease (PD), feature variable groups exist within the multimodal feature matrix. Traditional Lasso, which relies on l1 regularization, focuses solely on penalizing individual feature variables and overlooks group effects, thus limiting its efficacy in multimodal joint feature selection. To address this limitation, we replaced the Lasso method in Fusion1 with the Sparse Group Lasso (sgLasso) algorithm. This method incorporates both l1 and l2 constraints, addressing inter-group and intra-group sparsity, thus refining the joint feature matrix and isolating a higher quality feature subset (Simon et al., 2013).


(4) $${\left(\beta \right)}^{s\,g\,L\,a\,s\,s\,o}=\underset{\beta }{\arg\min}\frac{1}{2}\,{\left|y-\sum_{l=1}^{L}X^{\left(l\right)}\,\beta^{\left(l\right)}\right|}_{2}^{2}+\lambda_{1}\,\sum_{l=1}^{L}{\left|\beta^{\left(l\right)}\right|}_{2}+\lambda_{2}\,{\left|\beta \right|}_{1}$$

Among them, parameter *λ*_1_ is used to adjust the inter-group sparsity, and parameter *λ*_2_ is used to adjust the intra-group sparsity.

The sgLasso method was incorporated into the hybrid feature selection process named Fisher-EnsgLasso-PLS, and forming a new feature fusion strategy referred to as Fusion2.

#### 2.4.2 Decision level fusion

**FIGURE 3:**
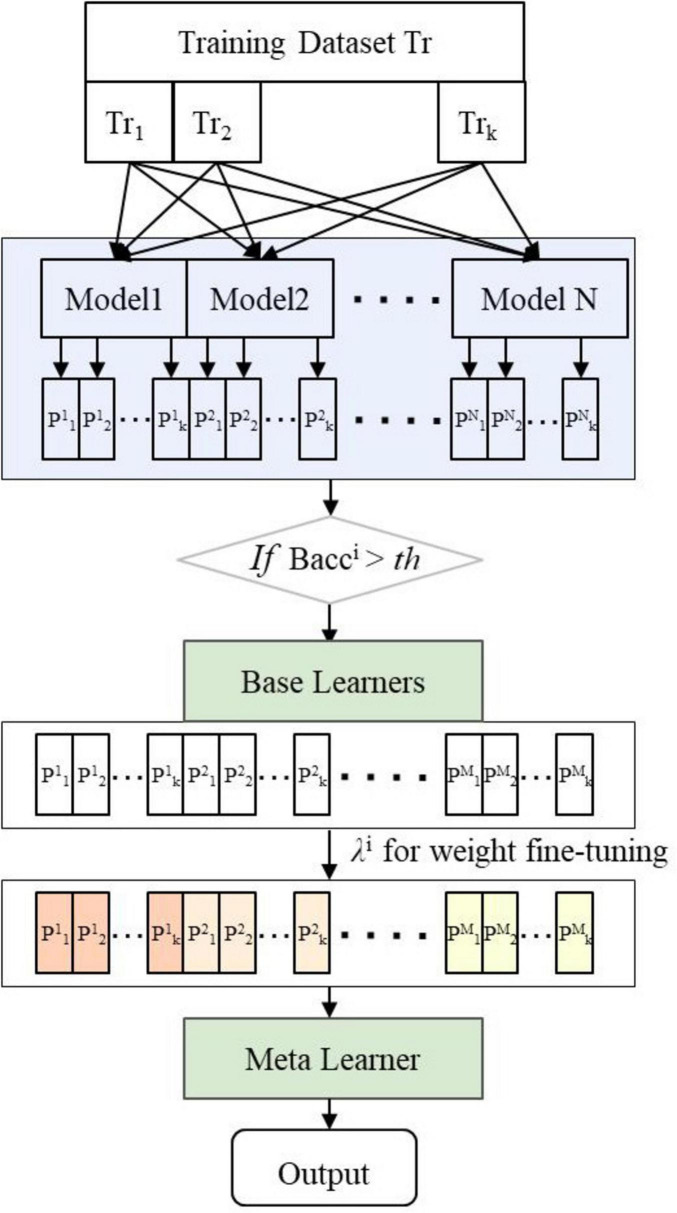
Framework of adaptive-selection-enhanced stacking ensemble learning (AE_Stacking).

The decision-level multimodal fusion method trains each modality separately to create N base classification models, then use ensemble learning to combine their predictions for a final result. The Stacking ensemble approach, ground in the principle of “collective intelligence” (Naimi and Balzer, 2018), merges outputs from diverse models using a meta-learner, enhancing classification accuracy and robustness beyond individual models. However, the choice of primary base classifiers is often subjective and not automated. We introduced an Adaptive-Selection-Enhanced Stacking ensemble learning framework (AE_Stacking), depicted in Figure 3. This framework adaptively selects*M* (*M* ≤ *N*)top-performing models from *N*base models and assigns weights based on their initial performance (see the GitHub repository).^5^ This enhances model diversity and representation, boosting overall integration performance. The implementation process is as follows:

##### 2.4.2.1 Step 1: data division

The training data is denoted as *T_r_*, and the test data as *T_e_*. Training set samples are divided into K subsets *T*_*r*_{*T*_*r*1_,*T*_*r*2_,…,*T*_*rk*_} via K-fold cross-validation. The first-layer base classifiers are represented as *C*_*layer*1_ {*C*_1_, *C*_2_,…, *C*_*N*_}, and the second-layer meta-learner model as *Clayer* 2;

##### 2.4.2.2 Step 2: training first-layer base classifiers

Each base classifier *Ci* is trained using K-1 folds, making predictions on the remaining fold to obtain predicted probability values for each model.

##### 2.4.2.3 Step 3: adaptive selection and boosting

The balanced accuracy $B\,a\,c\,c_{k}^{i}$ for each base classifier is calculated on K-fold training samples. Base models with *Bacc* exceeding a predefined threshold τ are selected as the first-layer base learners.


(5) $$B\,a\,c\,c=\frac{1}{2}\,\left(\frac{T\,P}{T\,P+F\,N}+\frac{T\,N}{T\,N+F\,P}\right)$$

(6) $${\overset{⌢}{C}}_{i}=\{\begin{array}{c} C_{i},\;Bacc^{i}\;>\;\frac{1}{N}\;\sum_{i=1}^{N}\left(\frac{1}{K}\sum_{k=1}^{K}Bacc_{k}^{i}\;\right)\\ reject,otherwise\; \end{array}$$

where TP, TN, FP, and FN correspond to true positives, true negatives, false positives, and false negatives, respectively.

The prediction probability scores of the selected*M* base models on the training samples *T*_*rk*_ are concatenated to obtain initial prediction results *P*{*p*^1^,*p*^2^,…,*p^M^*}. Additionally, weights λ*^i^*for the selected base learner are computed according to their $B\,a\,c\,c_{k}^{i}$:


(7) $$\mu^{i}=\frac{1}{M}\,\sum_{i=1}^{M}B\,a\,c\,c_{k}^{i}$$

(8) $$\lambda^{i}=-\frac{1}{M}•\frac{1}{\left(1-\frac{1}{{\left(\mu^{i}\right)}^{2}}\right)}$$

The weight parameter λ*^i^* fine-tunes each base learner, enhancing integration performance.

##### 2.4.2.4 Step 4: meta-learner training and prediction

The weighted initial predictions from Step 3 (*P*{*p*^1^,*p*^2^,…,*p^M^*}) serve as inputs for training the second-layer learner. Each base learner makes K predictions on the test set *T_e_* during the first-layer.

The initial prediction result for each base learner (*Q*{*q*^1^,*q*^2^,…,*q^M^*}) is obtained by weighted averaging the K test results, which is then multiplied by λ*^i^* to form the new test set for the meta-learner model. The meta-learner’s performance on this new test set determines the final results.

### 2.5 Modeling and evaluation

In this study, we employed eight well-established ML classification algorithms: Logistic regression (LR), Support Vector Machine (SVM), multilayer perceptron (MLP), Adaptive Boosting (AdaBoost), Random forest (RF), Gradient boosting decision tree (GBDT), Extreme gradient boosting (XGBoost) and Light gradient boosting machine (LightGBM). These algorithms were utilized to develop binary classification models for differentiating between PD and HC, followed by a comparative analysis of their performance.

#### 2.5.1 Modeling

To evaluate the efficacy of multimodal fusion of SNPs and MRI data in enhancing PD classification performance, we implemented two distinct strategies:

##### 2.5.1.1 Strategy 1: feature-level multimodal fusion classification

We compared the classification performance of three different input models—MRI, SNP, and their combination—using eight classifiers. For the fusion of MRI and SNP features, we evaluated Fusion1 and Fusion2 method. Hyperparameter optimization ranges for each classifier are in Supplementary Table S1, with tuning done via nested five-fold cross-validation and grid search.

#### 2.5.1.2 Strategy 2: decision-level multimodal fusion classification

In this strategy, the aforementioned eight classifiers served as candidate base classifiers within our proposed AE_Stacking framework. This framework automatically selects the optimal base classifier for ensemble combination and feature enhancement. A simple logistic regression model was employed as the meta-learner to mitigate the risk of overfitting. To demonstrate the superiority of decision-level multimodal fusion of imaging and genetic features, we conducted several comparative experiments: (1) comparison against single-modality single-classifier performance; (2) comparison with single-modality multi-classifier performance, where the ensemble model’s input consisted solely of single-modality features, assessing performance solely based on multi-classifier fusion; and (3) comparison with the feature-level fusion model.

#### 2.5.2 Evaluation

The dataset was hierarchically divided into training, validation, and testing sets at a ratio of 8:1:1. To ensure fairness and address the limitations of sample size, the data was randomly shuffled, and the above steps were repeated ten times, resulting in ten distinct sets of training, validation, and test sets. The training set was used for model training, the validation set for optimal feature subset determination and hyperparameter tuning, and the test set for recording the balanced accuracy (Bacc), sensitivity (Sen), specificity (Spe), G-mean, F1-score, and AUC values as classification performance metrics. The mean and standard deviation (mean ± SD) of the results from ten runs were reported for performance comparison between models.

### 2.6 Statistical analysis

SPSS (IBM SPSS 26.0, SPSS Inc.) software was used for statistical analyses. For the comparison of demographic variables, the chi-square test (χ^2^-test) was used to assess the differences in sex and t test was performed for comparisons of age. The Mann–Whitney U test was used to compare non-normally distributed data. All statistical tests were two-tailed, and *p* < 0.05 was considered significant.

## 3 Results

### 3.1 Clinical information

The demographic, clinical, and neuropsychological characteristics of 113 patients with PD and 54 HC are summarized in Table 1. The groups exhibited no significant differences in age, sex, or education level (*p* > 0.05). Significant differences in clinical non-motor symptoms were observed in the MDS-UPDRS-III, UPSIT, and SCOPA-AUT scales (*p* < 0.0001) across all PD and HC participants.

**TABLE 1:** Demographics, clinical and neuropsychological characteristics of study participants.

	**PD (*n* = 113)**	**HC (*n* = 54)**	***p*-value**
Sex (male/female)	66/47	33/21	0.626
Age (years)	61.02 ± 8.74	62.23 ± 8.05	0.345
Education level (years)	15.41 ± 3.02	15.37 ± 2.97	0.859
Hoehn & Yahr	1.61 ± 0.49	N/A	–
MDS-UPDRS-III	21.04 ± 9.00	0.50 ± 1.27	<0.001**
UPSIT	22.13 ± 8.61	33.30 ± 4.71	<0.001**
GDS	4.60 ± 1.78	4.77 ± 1.82	0.479
MoCA	27.71 ± 2.02	28.24 ± 1.09	0.304
SCOPA-AUT	9.62 ± 6.67	6.24 ± 4.58	<0.001**

Data are presented as means (standard deviation). PD, Parkinson’s disease; HC, health control; MDS-UPDRS-III, Part three of the Unified Parkinson Disease Rating Scale; UPSIT, University of Pennsylvania Smell Identification Test; MoCA, Montreal Cognitive Assessment; GDS, the Geriatric Depression Scaled; SCOPA-AUT, the Scales for Outcomes in PD Autonomic; χ^2^-test was performed for comparisons of sex; *t* test was performed for comparisons of age; Mann-Whitney U test was applied to compare non-normal distribution data;

**p < 0.001; *p < 0.05.

### 3.2 Classification performance of SNP

This study first evaluated the classification performance for PD using single-modality SNP genotype data, and validated the efficacy of the Fisher-EnLasso-PLS feature selection method through ablation studies. The integration of genetic and imaging data risks reducing sample size and introducing bias. To address this, experiments were conducted on a dataset comprising 532 subjects (367 PD patients and 165 healthy controls). A linear kernel SVM with L1 regularization was trained, validated, and tested on the selected features. Table 2 presents the average classification performance and the optimal number of features derived from ten experimental runs per method.

**TABLE 2:** Ablation comparison experiments.

**Feature selection method**	**Average number of features**	**Bacc**	**G-mean**	**F1**
Fisher	313	86.55 ± 6.62	85.95 ± 6.99	82.79 ± 8.11
Fisher-EnLasso	259	87.53 ± 6.11	87.07 ± 6.22	83.84 ± 7.02
Fisher-PCA	46	89.63 ± 5.44	89.45 ± 4.85	85.83 ± 7.49
Fisher-PLS	16	90.05 ± 4.76	90.00 ± 5.45	85.18 ± 6.78
Fisher-EnLasso-PCA	27	91.61 ± 3.40	91.41 ± 3.60	89.04 ± 3.98
Fisher-EnLasso-PLS	9	94.54 ± 2.17	94.50 ± 2.20	92.36 ± 2.40

The Fisher-EnLasso-PLS hybrid feature selection method demonstrated superior performance, achieving the highest accuracy with fewer features. It attained an average balanced accuracy (Bacc) of 94.54%. The EnLasso stage, following Fisher selection, effectively eliminated redundancies and reduced dimensionality, while EnLasso alone only marginally improved performance, increasing average Bacc by 0.98% compared to the Fisher-only method.

**FIGURE 4:**
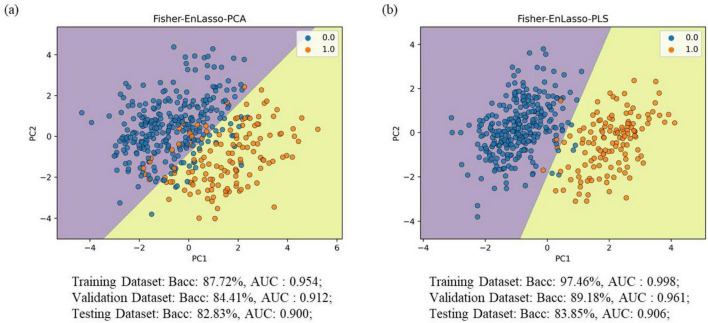
Visualization of the data (training set) distribution after Fisher-EnLasso feature selection. **(A)** The visualization result using the PCA algorithm. **(B)** The visualization result using the PLS algorithm; decision dividers are plotted based on the SVM model. “0” indicates PD patient samples; “1” denotes HC control samples.

In comparisons between Fisher-PCA and Fisher-PLS, the classification performance was inferior to the Fisher-EnLasso-PCA and Fisher-EnLasso-PLS techniques, underscoring the EnLasso algorithm’s efficacy in removing unnecessary complex features. Moreover, PLS proved more effective than PCA in dimensionality reduction. The Fisher-EnLasso-PLS algorithm, utilizing cascaded PLS, achieved a 2.93% higher average accuracy than Fisher-EnLasso-PCA with cascaded PCA, along with a narrower standard deviation. To illustrate performance differences, PCA and PLS were used to reduce the feature space to two dimensions after Fisher-EnLasso selection, visualizing data distribution and SVM (linear kernel, l1 = 0.01) decision boundaries in the initial experiment. Figure 4 shows that PLS-extracted principal components more effectively distinguished between positive and negative samples than PCA.

### 3.3 Classification performance of SNP and MRI fusion

#### 3.3.1 Feature-level fusion classification outcomes

The effectiveness of classification using feature-level integration of SNP and MRI data is detailed in Table 3. Key findings include: (i) Single SNP features consistently exhibited superior classification capability compared to MRI imaging features; (ii) The Fusion2 method generally achieved higher accuracy than Fusion1, surpassing the best performance of individual modalities. For instance, Fusion2’s accuracy, combining image and genetic data, exceeded the top-performing MLP model by 8.49% for MRI features and by 1.8% for SNP features; (iii) Multimodal fusion did not universally outperform unimodal approaches. Specifically, the SNP-based model outperformed multimodal fusion when using RF, AdaBoost, and GBDT classifiers, potentially due to suboptimal “learning” of MRI features, which could introduce noise and diminish classifier performance.

**TABLE 3:** Classification performance based on multi-modal fusion features and single-modal features.

**Classifier**	**MRI**	**SNP**	**Fusion1**	**Fusion2**
LR	78.72 ± 8.71	87.15 ± 5.51	85.84 ± 5.11	87.72 ± 4.39
SVM	80.68 ± 11.36	85.51 ± 9.33	84.93 ± 8.89	89.13 ± 7.56
RF	70.64 ± 11.63	87.00 ± 8.86	78.77 ± 10.68	72.92 ± 9.77
MLP	83.60 ± 7.04	90.29 ± 6.48	89.64 ± 5.57	92.09 ± 3.91
AdaBoost	74.19 ± 9.59	85.67 ± 9.67	73.43 ± 8.37	76.40 ± 7.09
GBDT	72.08 ± 7.01	84.29 ± 8.41	73.93 ± 8.50	76.57 ± 8.21
XGBoost	81.31 ± 10.79	87.27 ± 6.82	85.18 ± 6.76	88.73 ± 5.79
LightGBM	78.04 ± 10.77	84.48 ± 5.41	83.54 ± 8.48	85.57 ± 4.54

The values in bold represent the best mean accuracy achieved in each feature fusion model.

#### 3.3.2 Decision-level fusion classification results

To demonstrate the effectiveness of decision-level fusion with the AE_Stacking technique for integrating image and genetic features, ablation studies were conducted. This method was benchmarked against the best single-modal model with one classifier, single-modal models with multiple classifiers, and feature-level fusion models. As shown in Table 3, MLP emerged as the top performer for both unimodal and feature-level fusion, serving as the primary benchmark. We also tested the single-modal multi-classifier approach by inputting each modality’s features into AE_Stacking and adjusting the multi-classifier configuration. Additionally, we evaluated a model trained on a multimodal feature matrix with feature-level fusion as input to AE_Stacking. The data in Table 4 revealed that the AE_Stacking decision-level fusion achieved the highest average balanced accuracy, sensitivity, G-mean, and F1 scores-95.36, 94.36, 95.30, and 93.41%, respectively, surpassing feature-level fusion (*p* < 0.05). Moreover, AE_Stacking models with integrated multi-classifiers consistently delivered improved classification outcomes, characterized by a high mean and a reduced standard deviation (*p* < 0.05), when compared to single classifier models, irrespective of whether unimodal or feature-level multimodal fusion was utilized. This underscores both the efficacy and stability of the proposed strategy.

**TABLE 4:** Classification performance based on decision-level fusion.

	**MRI**		**SNP**		**Fusion2**		**MRI+SNP**	
	**MLP**	**AE_Stacking**	**MLP**	**AE_Stacking**	**MLP**	**AE_Stacking**	**MLP**	**AE_Stacking**
Bacc	83.88 ± 9.30	85.27 ± 5.66	90.29 ± 6.48	91.09 ± 6.15	92.09 ± 5.80	93.27 ± 5.57	–	95.36 ± 4.77
Sen	78.67 ± 20.50	76.00 ± 14.97	84.36 ± 13.73	84.00 ± 13.63	86.00 ± 12.72	92.00 ± 9.80	–	94.36 ± 7.37
Spe	89.09 ± 10.60	94.55 ± 9.92	97.35 ± 3.50	96.36 ± 6.80	98.18 ± 3.64	94.55 ± 7.27	–	96.36 ± 3.40
G-mean	82.19 ± 12.91	84.48 ± 7.15	90.29 ± 7.95	89.71 ± 7.41	91.55 ± 6.14	93.03 ± 5.95	–	95.30 ± 4.45
F1	77.05 ± 6.18	80.45 ± 4.45	88.23 ± 9.36	87.43 ± 8.64	90.14 ± 8.30	90.44 ± 6.37	–	93.41 ± 4.81

**FIGURE 5:**
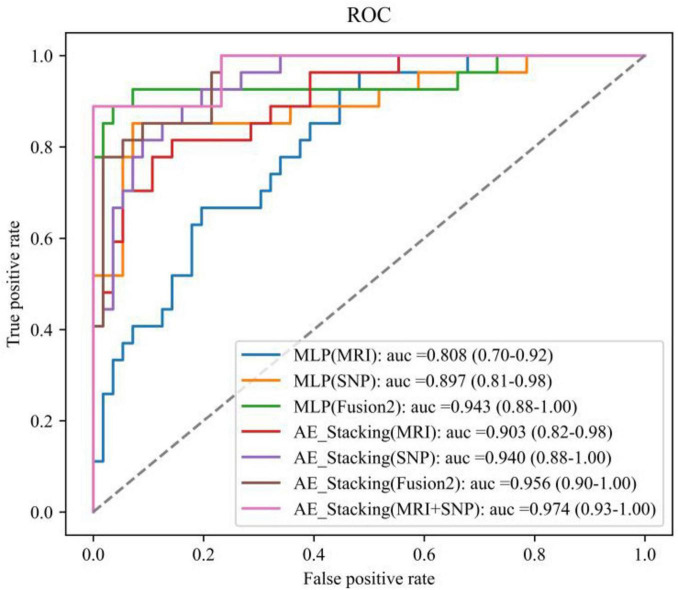
ROC curves for each model.

Figure 5 presents the ROC curves and AUC metrics for each model, with 95% confidence intervals indicated in parentheses. The AE_Stacking-based multimodal fusion model notably achieved the highest AUC of 0.974, with a 95% confidence interval spanning from 0.93 to 1.00.

### 3.4 Cerebral regions and genetic markers associated with PD

**FIGURE 6:**
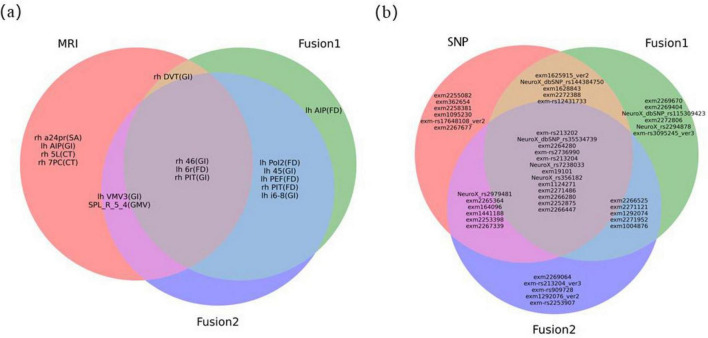
Venn diagram of optimal features. **(A)** the optimal MRI image feature. “lh” represented regions located in the left cerebral cortex, while “rh” represented regions located in the right cerebral cortex. The characters in parentheses indicated the feature parameter index for each brain region. For detailed information on brain anatomy and definitions of brain regions, it could refer to websites http://www.brainnetome.org/ and http://www.humanconnectome.org/; **(B)** the optimal SNPs gene features.

The weights of each feature in ten experiments were averaged and then ranked in descending order to determine their relative contributions for PD. For MRI image features, we identified the top 10 ranked brain regions in each method. Due to the high dimensionality of the genetic data, we reported the top 30 ranked SNP loci. Figure 6 illustrates the Venn diagrams of brain regions and genes identified as the best features by unimodal and multimodal methods.

**FIGURE 7:**
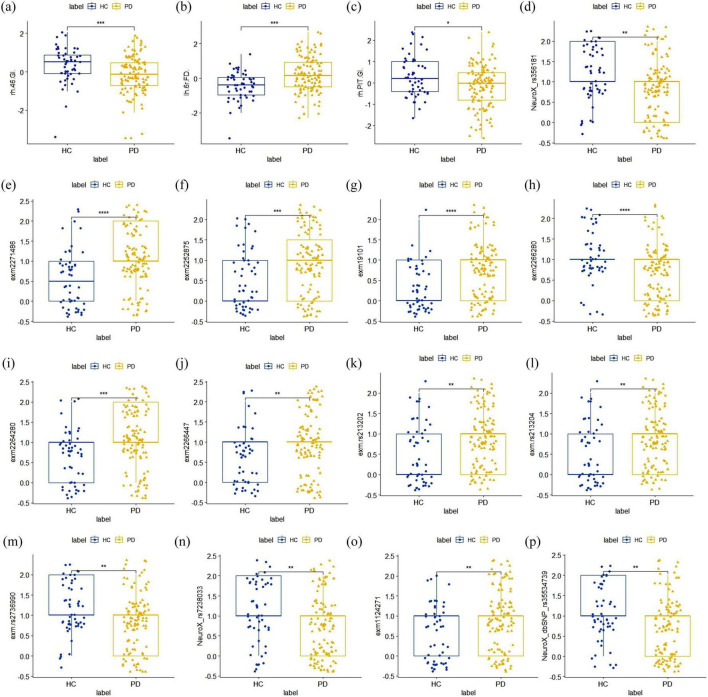
Distribution plot of inter-group differences in the optimal stable features. **(A–C)** The optimal MRI image feature. Compared to the HC group, the lh 6r(FD) feature showed a significant increase in the PD group, while the rh 46(GI) and rh PIT(GI) features both exhibited significant atrophy; **(D–P)** the optimal SNPs features. All of these SNPs showed statistically significant differences between the PD group and the HC group.

Among MRI features, the FD feature corresponding to the 6r brain region in the left cerebral cortex [lh 6r (FD)], the GI feature corresponding to the 46 brain region on the right [rh 46 (GI)], and the right posterior inferotemporal region [rh PIT (GI)] consistently appeared in the top 10 features across all three sets of experiments. This indicates that they are the most stable imaging features with strong discriminative power for PD in MRI modality. The location of the these three regions in the brain were shown in Supplementary Figure S2. Compared to the HC group, the lh 6r (FD) feature showed a significant increase in the PD group, while the rh 46 (GI) and rh PIT (GI) features exhibited significant atrophy (Figures 7A–C).

For SNP loci, we referenced the international 1,000 genomes project (1,000 genomes) for gene annotation of the stable SNP loci ranked in the top 30 and reviewed relevant literature. As shown in Table 5, the SNPs with high discriminatory power were mainly located in the SNCA gene, the coding region of the VPS52 gene, and the SLC14A1 gene, all of which have been confirmed to be associated with PD. These SNPs demonstrated statistically significant differences between the PD and HC groups (Figures 7D–P).

**TABLE 5:** TOP30 SNPs and corresponding gene annotations.

**SNPs**	**Rs code**	**Chromosome location**	**Genes**	**Literature coverage**
NeuroX_rs356181	rs356181	chr4: 90626111	SNCA	Blauwendraat et al. (2020), Jia et al. (2022), and Sampedro et al. (2018)
exm2271486	rs7939948	chr11:36311014	COMMD9	–
exm2252875	rs1131636	chr17:1801189	RPA1	Yuan et al. (2020)
exm19101	rs486557	chr1:15616139	FHAD1	–
exm2266280	rs1588787	chr6:163354979	PACRG	Liu et al. (2021)
exm2264280	rs7015855	chr8:13660787	–	–
exm2266447	rs12704649	chr7:92674917	–	–
exm-rs213202	rs213202	chr6:33232055	VPS52	Wei et al. (2023)
exm-rs213204	rs213204	chr6:33241076	VPS52	Wei et al. (2023)
exm-rs2736990	rs2736990	chr4:90678541	SNCA	Blauwendraat et al. (2020); Jia et al. (2022), and Sampedro et al. (2018)
NeuroX_rs7238033	rs7238033	chr18:43316966	SLC14A1	Recabarren and Alarcon (2017)
exm1124271	rs57740714	chr14:94912896	SERPINA11	–
NeuroX_dbSNP_rs35534739	rs113343065	chr6:32583490	HLA-DQA1	Wissemann et al. (2013) and Yu et al. (2021)

“–” indicates that no related genes and related literature reports were found.

## 4 Discussion

The purpose of this study is to establish a multi-modal fusion learning model that enhances the diagnostic capabilities for PD by integrating MRI image data and genetic information. Our findings indicate that the decision-level fusion model (AE_Stacking) achieved the highest accuracy (95.36%) and AUC (0.974) in identifying PD, significantly outperforming models based solely on imaging or genetic data. Furthermore, the decision-level fusion model demonstrated superior performance compared to feature-level fusion models. By analyzing the latent features extracted by the model, we identified risk factors highly correlated with the disease, providing new insights into PD diagnosis.

Numerous studies have utilized ML or deep learning algorithms to investigate the value of various data modalities in PD diagnosis, such as clinical assessment scales (Araújo et al., 2023; Vitorio et al., 2023), electroencephalographic monitoring (Nour et al., 2023; Suuronen et al., 2023), cerebrospinal fluid biomarker detection (Höglinger et al., 2024; Tsukita et al., 2023), neuroimaging, and genetic testing. However, clinical assessment scales are often subjective, lacking adequate sensitivity and specificity; cerebrospinal fluid collection is invasive; and electroencephalographic monitoring is heavily influenced by individual variability, struggling to reflect changes in deeper brain regions. Consequently, these methods face considerable challenges in early PD screening. Imaging genomics represents an emerging field in data science. Neuroimaging genomics enable the integrated analysis of brain imaging and genomic data, providing new insights into brain phenotypes, genetic, and molecular characteristics and their influence on both normal and disordered brain functions and behaviors. For example, the G/A polymorphism may cause more extensive brain white matter damage in PD (Yu et al., 2022). Early-onset Parkinson’s disease with atypical molecular imaging abnormalities in a patient carrying the de novo PRKCG mutation (Chen et al., 2022). Key genetic signatures of large-scale PD pathology have contributed to focal neuronal vulnerability to disease progression (Basaia et al., 2022). Therefore, exploring the underlying causes of PD from the source can provide more reliable and accurate foundations for PD diagnosis.

Effectively mining the associative information between imaging and genetic data poses a considerable challenge. Unlike the work of Bi et al. (2021a,b), they used correlations of gene and brain functional information from fMRI as model inputs. Although high accuracy rates (88.57%) were achieved, solely relying on fused features risked obscuring unique modality-specific information, potentially diminishing the model’s overall performance by not fully leveraging the distinct contributions of each data type. To overcome these limitations, we developed and compared two fusion methods: feature-level and decision-level fusion. As shown in Table 4 and Figure 5, compared with the feature-level fusion method, the model using the designed AE_Stacking ensemble learning method for decision-level fusion achieved the highest classification accuracy (95.36%) and AUC value [0.974 (95%CI: 0.93–1.00]. In the feature-level fusion approach, the direct concatenation of SNP features with MRI features fails to facilitate meaningful interaction between disparate modal features. This limitation hinders the model’s ability to learn disease-related features from the fusion data. Conversely, the decision-level fusion strategy is similar to multi-task learning. By treating the two modal features as independent inputs, the model can perceive the learning process of each modality as a distinct task. Consequently, this fusion strategy effectively leverages both the commonalities and differences among various modalities to integrate the features of each modality comprehensively. The main advantage of using the AE_Stacking decision-level fusion strategy is the integration of the results of multiple strong classification base models. This method not only considers the independent contribution of each single modality feature to PD diagnosis, but also achieves complementary advantages of multiple base models through a “strong combination” approach, so the overall model has higher PD recognition performance. In addition, compared with the general Stacking integration technology, the designed AE_Stacking integration learning method can automatically select models with better classification performance from the candidate classifiers as strong base classifiers, thereby enhancing the diversity of base classifiers. Then increase the weight of the base learner with high classification performance, and reduce the weight of the base learner with relatively low classification performance, so that the meta-learner can pay more attention to the base learners with stronger performance. Compared with direct input, the AE_Stacking method improves performance by automatically selecting the best performing base learners, thereby increasing diversity and allowing the meta-learner to focus on more powerful models, and therefore enhancing integration performance. Future research could be integrated with more multimodal data to develop comprehensive predictive models.

Finally, by statistically analyzing the features selected for high frequency in each single-modal as well as two multimodal feature fusions process, we identified a subset of stable brain region features that consistently belonged to the distinguished circle of the top 10 features across the entirety of our experimental datasets. The FD attribute pertaining to the left cerebral cortex’s 6r region [lh 6r (FD)] and the GI attribute linked to the right hemisphere’s 46 region [rh 46 (GI)] demonstrated remarkable stability, suggesting their potential as robust imaging biomarkers for PD. Interestingly, the most discriminative features in our model were based on advanced measurements of cortical geometry—specifically FD and GI—rather than traditional metrics like GMV, CT, and SA. This suggests that cortical surface geometry measurements may provide more sensitive biomarkers for PD diagnosis. This observation suggests that measurements of cortical surface geometry may offer more sensitive biomarkers for the diagnosis of PD. The heightened discriminative power of FD and GI could be attributed to their intrinsic sensitivity to the nuanced changes in cortical structure that are often subtle or overlooked by traditional volumetric methods. Specifically, fractal dimension captures the complexity and intricacy of the cortical folding patterns, while the gyrification index quantifies the degree of cortical folding, providing insights into the brain’s structural integrity and potential deviations caused by pathological processes. In addition, the changes observed in these cortical features are also reflect the combined effects of GM, WM, and the overall dynamics of cortical connectivity. Together, these elements provide a microscopic view of the structural alterations in the brains of individuals with PD.

Our genetic probe into SNP data unveiled that the most discriminative loci were predominantly situated within three genes: SNCA, with SNPs rs356181 and rs2736990; VPS52, with SNPs rs213202 and rs213204; and SLC14A1, with SNP rs7238033. The SNCA gene, in particular, has been recognized as an autosomal dominant culprit in PD (Jia et al., 2022). The rs356181 variant within the SNCA locus has been found to regulate the influence of cerebrospinal fluid-related biomarkers on cortical atrophy and is associated with diminished cognitive function in PD patients (Sampedro et al., 2018). The PACRG gene has been reported to be a bidirectional promoter shared with the Parkin/Park2 gene (Liu et al., 2021). The SLC14A1 gene exhibits altered expression in PD-affected individuals and is conjectured to be instrumental in the regulation of Aβ production and apoptosis, potentially contributing to the pathological hallmarks of Alzheimer’s disease, PD, and muscular dystrophy (Recabarren and Alarcon, 2017). Genetic variation can affect the corresponding cell function, thereby changing the normal brain phenotype. Therefore, combining genes with brain imaging will help understand the abnormalities of brain structure and function and the role of gene expression risk factors in the progression of PD (García-Marín et al., 2023; Zang et al., 2023). In the future, clinical symptoms should be further combined to explore the association between genotype, brain phenotype and clinical phenotype through joint analysis of fusion imaging genes. In addition, the combination of machine learning or deep learning technology is expected to predict PD risk and possible clinical manifestations at the individual level. The identification of these stable imaging biomarkers and genetic loci holds significant promise for personalized treatment strategies.

While our methodology enhances PD diagnostic efficacy, it is essential to acknowledge its limitations. The PLS method, used for dimensionality reduction, may restrict analysis and interpretation of important fusion features. Moreover, our study’s focus was specifically on the integration of genetic and MRI data for PD identification. While this approach has provided valuable insights, it also presents a limitation in terms of the breadth of data types considered. Future research should aim to expand the scope by incorporating additional imaging modalities, such as positron emission tomography (PET) or functional MRI (fMRI), which could offer different perspectives on the neurobiological changes associated with PD. In addition, genetic factors exhibit variation across different ethnic cultures. Ethnic heterogeneity is a further key determinant, influencing disparities in epidemiology, clinical manifestations and mortality. Therefore, it becomes necessary to take into account ethnic-cultural factors and more clinical manifestations to develop models that are both specific and sensitive to the unique genetic profiles present in diverse population. Finally, a growing number of genetic studies have demonstrated that there are disparities in the genetic mechanisms across different PD syndromes as well as in the severity of the disease (Koziorowski et al., 2021; Martínez Carrasco et al., 2023). This suggests that in the future, the multimodal fusion strategy of imaging and genetic data can also be applied to the differential diagnosis of PD and the evaluation and prediction of disease severity, thereby enhancing the accuracy and reliability of diagnosis and enabling the development of more refined and effective healthcare solutions.

## 5 Conclusion

In conclusion, the AE_Stacking model, trained with MRI and genetic data, demonstrates promising diagnostic values in detection of PD. By integrating multimodal data, the model has the potential to reveal complex patterns of disease progression. As the clinical sample size expands and data quality improves, this method is expected to be applied to more complex clinical tasks, such as staging disease progression, identifying high-risk populations, and supporting the differential diagnosis of related neurological disorders. Additionally, these consistent neuroimaging features and specific genetic variants identified highlight their potential as early diagnostic indicators for PD, while would also proffer new insights for personalized clinical interventions. Future research should focus on biological evaluations to demonstrate relevant neurobiological signals or markers and clarify the psychological or behavioral structures linked to specific brain pathways or regions, thereby strengthening the model’s reliability.

## Data Availability

The datasets presented in this study can be found in online repositories. The names of the repository/repositories and accession number(s) can be found in the article/Supplementary material.

## Footnotes

1
www.ppmi-info.org/dat

2
https://ida.loni.usc.edu/pages/access/geneticData.jsp

3
http://dbm.neuro.uni-jena.de/cat/

4
https://www.fil.ion.ucl.ac.uk/spm/

5
https://github.com/yyflib/Multimodal-ensemble-learning
