# Supplementary material for: Identification of Parkinson’s disease using MRI and genetic data from the PPMI cohort: an improved machine learning fusion approach
Source: Front Aging Neurosci. 2025 Feb 4;17:1510192. doi: 10.3389/fnagi.2025.1510192 (PMC11832485; doi:10.3389/fnagi.2025.1510192)
Supplement: Supplementary file 1 [file Table_1.docx]

**Supplementary Materials**

**Fig.S1.** **The flowchart of MRI image preprocessing and feature extraction.**

**Fig.S2. Location of brain regions exhibiting the highest discriminative ability and robust stability for PD.** The fractal dimension (FD) feature associated with the 6r brain region in the left cerebral cortex (lh 6r (FD)) (Left figure), which is located in the premotor cortex and is associated with motor functions. The gyrification index (GI) feature linked to the 46 brain region in the right hemisphere (rh 46 (GI)) and the right posterior inferotemporal region (rh PIT (GI)) (right figure). The 46 region, found in the inferior frontal cortex, plays a significant role in language functions, while the PIT region, located in the ventral visual cortex, is associated with visual processing. These brain regions consistently rank among the top ten features across all three experimental sets, thereby representing the most stable imaging characteristics with considerable discriminative power for PD within the MRI modality. “LH” , Left hemisphere of the brain, “RH” Right hemisphere of the brain. For detailed anatomical information and definitions of the brain regions, please refer to the brain template available at http://www.humanconnectome.org/ .

**Table S1. The hyperparameters and optimization range settings for each model.**

| Classifier | Hyperparameters and optimization range settings |
| --- | --- |
| LR | C (L2 regularization): [0.1,0.01,0.001,0.0001]; |
| SVM | kernel: “linear”;  C (L1 regularization): [0.1,0.01,0.001,0.0001]; |
| RF | max_depth: [3, 5, 8, 10, 12];  n_estimators: [50, 100, 150，200]; |
| MLP | hidden_layer_sizes: (10, 5);  activation: “ReLU”;  solver: “lbfgs”;  alpha: [0.1,0.01,0.001,0.0001]; |
| AdaBoost | Boosting algorithm: “SAMME.R”;  n_estimators: [50, 100, 150，200];  learning_rate: [0.01,0.001,0.0001]; |
| GBDT | n_estimators: [50, 100, 150，200];  learning_rate: [1，0.5，0. 1,0.01];  subsample: [1，0.5，0.3];  loss: “deviance”; |
| XGBoost | Booster: “gbtree”;  n_estimators: [100, 150，200];  max_depth: [3, 5, 8, 10, 12];  reg_lambda: [0.1，0.01，0.001];  learning_rate: [0.001，0.01，0.1]; |
| LightGBM | boosting_type: “gbdt”;  n_estimators: [100, 150，200];  max_depth: [3, 5, 8, 10, 12];  reg_lambda: [0.1，0.01，0.001];  learning_rate: [0.001，0.01，0.1]; |
